# U.S. COVID-19 vaccine distribution strategies, systems, performance, and lessons learned, December 2020 – May 2023

**DOI:** 10.1016/j.vaccine.2024.02.020

**Published:** 2024-02-15

**Authors:** Christopher Duggar, Jeanne M. Santoli, Cameron Noblit, Lori B. Moore, Roua El Kalach, Carolyn B. Bridges

**Affiliations:** a National Center for Immunization and Respiratory Diseases, Centers for Disease Control and Prevention (CDC), Atlanta, GA, United States; b General Dynamics Information Technology (GDIT) contractor supporting CDC’s COVID-19 Response, United States

**Keywords:** COVID-19 vaccine, COVID-19 pandemic, Vaccine distribution, Lessons learned, Emergency preparedness

## Abstract

During December 2020 through May 2023, the Centers for Disease Control and Prevention’s (CDC) Immunization Services Division supported and executed the largest vaccine distribution effort in U.S. history, delivering nearly one billion doses of COVID-19 vaccine to vaccine providers in all 50 states, District of Columbia, Puerto Rico, Virgin Islands, Guam, Federated States of Micronesia, American Samoa, Marshall Islands, Northern Mariana Islands, and Palau. While existing infrastructure, ordering, and distribution mechanisms were in place from the Vaccines for Children Program (VFC) and experience had been gained during the 2009 H1N1 pandemic and incorporated into influenza vaccination pandemic planning, the scale and complexity of the national mobilization against a novel coronavirus resulted in many previously unforeseen challenges, particularly related to transporting and storing the majority of the U.S. COVID-19 vaccine at frozen and ultra-cold temperatures.

This article describes the infrastructure supporting the distribution of U.S. government-purchased COVID-19 vaccines that was in place pre-pandemic, and the infrastructure, processes, and communications efforts developed to support the heightened demands of the COVID-19 vaccination program, and describes lessons learned.

## 1. Overview

From December 2020 through May 2023, the Centers for Disease Control and Prevention’s (CDC) Immunization Services Division supported and executed the nation’s largest vaccine distribution in U.S. history. Nearly one billion doses of frozen and refrigerated COVID-19 vaccine doses and ancillary kits, including syringes and vaccination cards, were delivered to COVID-19 vaccine program enrolled providers in all 64 U.S. immunization programs [all 50 states, District of Columbia, 5 cities (Chicago, Houston, New York City, Philadelphia, San Antonio), Puerto Rico, Virgin Islands, Guam, Federated States of Micronesia, American Samoa, Marshall Islands, Northern Mariana Islands (CNMI), and Palau)]. [1] In total 125, 000 jurisdiction provider sites, 54,000 individual pharmacy sites and 5,600 dialysis center sites were enrolled in the COVID-19 vaccine program and vaccine was shipped to over 107,500 unique locations by the end of the COVID-19 public health emergency declaration on May 11, 2023. [2] While existing infrastructure, ordering, and distribution mechanisms were in place from the Vaccines for Children Program (VFC) [3] and experience had been gained during the 2009 H1N1 pandemic and incorporated into influenza vaccination pandemic planning [4], the scale and complexity of the national mobilization against a novel coronavirus was beyond any imagined response. This scale and complexity resulted in many previously unforeseen challenges, particularly related to transporting and storing the majority of the U.S. COVID-19 vaccine which required a frozen (−50°C and − 15°C) or ultracold (−90°C and − 60°C) cold chain. CDC’s routine distribution of approximately 70–80 million doses of vaccine to a network of 38,000 VFC providers annually was rapidly eclipsed by the COVID-19 vaccine operations scale. [5] (Personal communication, Jeanne Santoli, CDC August 2023). Further, the group at highest risk for severe illness or death from SARS-CoV-2 infection included older adults, especially those living in nursing homes and other congregate care settings, necessitating the rapid development and deployment of vaccine distribution systems specifically for persons living in long-term care facilities (LTCF). [6] Importantly, as has been documented for seasonal influenza [7] and pneumococcal pneumonia hospitalizations, [8–9] the burden of SARS-CoV-2 hospitalization and complications was higher among Hispanic and non-Hispanic Black populations relative to non-Hispanic White populations. Higher social vulnerability, lower income, and work as front-line essential workers were also associated with higher risk of COVID-19. [10–13] Thus, targeted efforts were needed to ensure vaccine access in all areas. A federal pharmacy partnership was developed to distribute vaccines through existing pharmacy distribution systems to local pharmacies around the U.S., [14] and mechanisms were established with the Health Resources and Services Administration (HRSA) to distribute vaccines to federally qualified healthcare centers to ensure access for vulnerable populations.

In this article, we will describe, with a focus on CDC-led efforts, the challenges posed by COVID-19 vaccines in relation to prior pandemic planning and the 2009 H1N1 influenza pandemic experience, and the infrastructure supporting the distribution of U.S. government-purchased COVID-19 vaccines that was in place pre-pandemic. In addition, we describe the infrastructure, processes, and communications efforts developed to support the expanded impact and demands of the COVID-19 pandemic and the results of vaccine distribution efforts across the U. S. Finally, we provide a summary of lessons learned with regards to future planning of emergency response vaccine distribution efforts.

## 2. Introduction and Background

The COVID-19 vaccines and the pandemic response posed many challenges that were unforeseen and differed from the 2009 H1N1 influenza pandemic response, requiring new systems and infrastructure. In both 2009 H1N1 and COVID-19, vaccine development began rapidly even before a pandemic was declared. [15] And all COVID-19 vaccine doses were federally purchased and provided to the public at no out-of-pocket cost, similar to 2009 H1N1 influenza vaccines. [16] But very different from 2009 H1N1, where vaccines werelicensed by the U.S. Food and Drug Administration (FDA), none of the COVID-19 vaccines were initially licensed, and were instead available under an emergency use authorization (EUA); this required the use of patient and provider fact sheets to ensure informed use of the vaccine instead of Vaccine Information Statements. [17] Further, all vaccine providers were required to enroll as COVID-19 vaccine providers, even if they were already Vaccines for Children (VFC) providers which differed from 2009 H1N1. [23]

### 2.1. Vaccines for Children (VFC) Infrastructure

Similar to 2009 H1N1, planning for implementation of vaccine distribution for a nationwide emergency COVID-19 vaccine program started with the foundation of the VFC infrastructure, including CDC’s centralized vaccine distribution contract (awarded to McKesson Corporation before and during the COVID-19 response) which includes an option for the distribution of pandemic vaccines. The VFC program, managed by CDC, purchases vaccines for all eligible children in the United States (approximately half of all U.S. children are eligible). The goal of the VFC program is to ensure that all children, regardless of their ability to pay for vaccines, have a better chance of getting recommended vaccines. Vaccines recommended by CDC for children are distributed to enrolled providers at the direction of state and local immunization programs. Providers are enrolled by state and local immunization programs who approve vaccine allocations to providers and conduct quality assurance visits with providers, most of whom are pediatric providers. Providers receive doses of all routinely recommended vaccines at no cost and are not allowed to turn eligible children away because of an inability to pay a vaccine administration fee. However, providers may bill Medicaid for vaccine administration costs for children enrolled in Medicaid. As defined by the statute that created the VFC program, eligible children include those 18 years and younger who are uninsured, Medicaid eligible, American Indian or Alaska Native, or who are underinsured (i.e., are covered by an insurance policy that doesn’t cover any vaccines or doesn’t cover certain recommended vaccines). Underinsured children may only receive VFC vaccines at a federally qualified healthcare center (FQHC) or rural health clinic or under an approved deputization agreement. [18]

### 2.2. Pre-COVID-19 pandemic vaccine distribution planning assumptions and reporting needs

Before COVID-19, pandemic vaccine distribution planning was modeled on pandemic influenza and included incorporation of lessons learned from the 2009 H1N1 pandemic. [19] Assumptions included that, as occurred with other influenza pandemics, younger children and pregnant women would likely be at higher risk for illness and severe illness and would be prioritized along with healthcare personnel and other essential worker populations. [20] Other logistical assumptions included that vaccines would require refrigerator temperatures for shipping and storage, that minimum doses per order would be 100 doses, similar to 2009 H1N1, and vaccines would have clear shelf-life limits.

The lessons learned from influenza and pandemic response modeling highlighted the need to expand the number of vaccine providers to vaccinate the public as quickly as possible in future pandemics. Enrollment of pharmacists and additional medical vaccine providers for adults was identified as a critical need to support large-scale vaccination of all ages since most VFC providers are pediatric or family medicine providers. [21]

Obtaining accurate information on vaccine inventory at provider sites, doses administered and vaccine wastage were challenges during the 2009 H1N1 pandemic vaccine program given that reporting to immunization information systems was not required for 2009 H1N1 vaccine doses and, in general, utilization of Immunization Information Systems (IIS) by providers for adult vaccines was limited at that time. [22] Thus, information on 2009 H1N1 vaccine uptake was mostly limited to survey data, highlighting the need for improvements in vaccine dose tracking, including reporting of doses administered to IIS. [16]

### 2.3. COVID-19 Vaccine Distribution Challenges

In contrast to 2009 H1N1, COVID-19 vaccine distribution processes and systems needed major adjustments from manufacturers, distributors, public health, and providers due to requirements for some vaccine doses to be shipped in ultracold packaging with up to 975–1170 dose ordering minimums. No prior vaccination program had included vaccines that required ultracold shipping and storage. In addition, limited vaccine stability data for newly developed vaccines initially resulted in short shelf lives that were extended as additional stability data became available. [16] Ensuring appropriate cold chain for COVID-19 vaccines shipped and stored at ultracold temperatures necessitated establishing sources for dry ice supplies and specialized packing. Moreover, managing anticipated changes in shelf-life required providers to use online tools to check expiration dates for the most current and accurate shelf-life information, an additional step that contributed to the complexity of vaccine inventory management. [16]

## 3. COVID-19 Vaccine Providers and Vaccine Ordering, Distribution and Reporting

To address the need for rapid vaccination of the U.S. population, particularly older adults in congregate settings, and to ensure equitable access for vulnerable populations, groups of providers enrolled to receive COVID-19 vaccine included:

1. Private medical and pharmacy providers, including hospitals and outpatient clinics
2. State and local health departments for public health clinics
3. Federal retail pharmacy program participants, [14] some of whom also participated in early vaccination of residents and staff in LTCF [23]
4. Federal entities, including Health Resources and Services Administration (HRSA) which worked with federally qualified healthcare centers (FQHC), Veteran Health Administration (VHA), Bureau of Prisons (BOP), Indian Health Service, Department of Defense (DoD), National Institutes of Health, and Department of State
5. Two national renal dialysis chains (added approximately 5 months into the vaccination program)

**Fig. 1.**
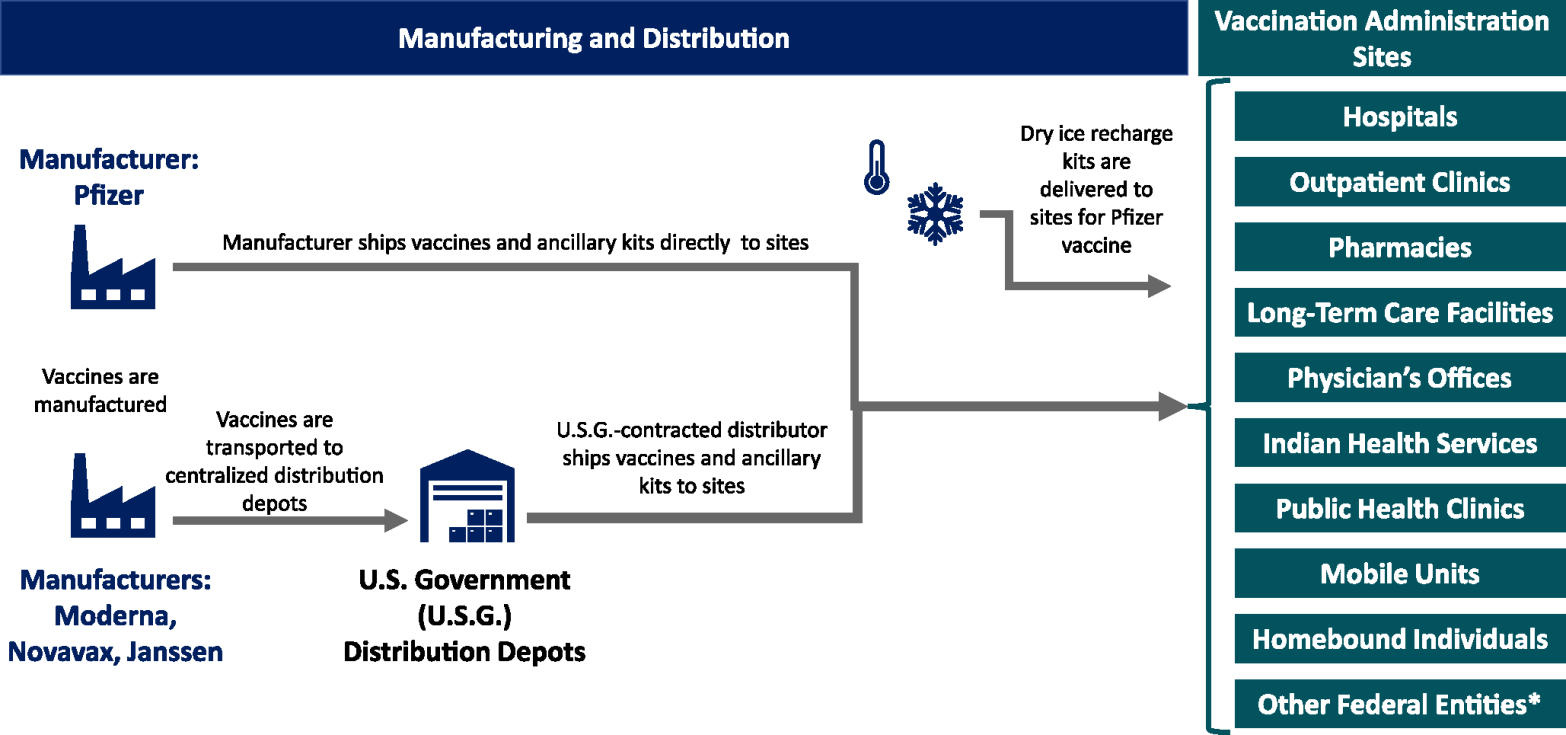
Federal COVID-19 Response Vaccine Distribution Process Footnote: *Includes federally qualified health centers, Veteran Health Administration, Bureau of Prisons, Department of Defense, National Institutes of Health, and Department of State.

Enrolled providers administered vaccines in a wide range of settings, including large chain and independent pharmacies, public health and private practice clinics, Indian Health Service clinics, mobile clinics, hospitals, long term care facilities, dialysis centers, and at homes of homebound persons (Fig. 1). Most providers were enrolled through their state or local departments of health. To improve tracking of vaccine administered and inventory in providers’ possession, providers were required to enroll in and then report vaccine administration to their jurisdiction’s IIS daily within 24–72 h of vaccine administration. Providers were also required to report inventory remaining and expired doses to Vaccines.gov (a web-based system formerly called Vaccine Finder) initially on a daily basis then on a weekly basis. [24] Vaccines.gov also allowed providers the option of having their location displayed on the website as one that offered COVID-19 vaccines to the public. Locations with COVID-19 vaccine available to be administered to the public were searchable by zip code and displayed on a map, which helped the public identify COVID-19 vaccination locations convenient to them. [25]

Census data and vaccine availability projections were used to determine how much vaccine would be made available to each jurisdiction. State and local health departments then determined the number of doses to be distributed to each of the enrolled providers in their state, while CDC worked with commercial program partners and federal entities to determine their vaccine allocations.

The Vaccine Tracking System (VTrckS), CDC’s vaccine order management system, which supports routine vaccination ordering and distributing of 70–80 million doses of vaccine annually, was utilized as the platform for COVID-19 vaccine ordering. VTrckS users (62 state, local, and territorial public health jurisdictions) and enrolled national provider organizations [(i.e., the Department of Veterans Affairs (VA), DoD, Indian Health Service (IHS), BOP, 21 pharmacy organizations (including large retail chains, coordinating pharmacy services administrative organizations (PSAOs) representing independent retail and long-term care pharmacies, and pharmacy network administrators), and 2 national dialysis partners participating in COVID-19 vaccination)] utilized VTrckS to:

- Manage enrollment of providers to the COVID-19 vaccination program
- Onboard provider sites to the program
- Manage and update provider addresses, shipping information, and other details
- Assess and manage vaccine allocations
- Place and approve vaccine orders for provider sites
- Verify provider’s compliance with wastage reporting requirements
- Track vaccine shipment
- Generate end-to-end distribution reports

As part of the overall IT infrastructure to support COVID-19 vaccination, VTrckS leveraged the VTrckS External Information (ExIS) System Portal to exchange data with partners. Examples of partner systems actively exchanging COVID-19 data throughout the response include:

- Jurisdictions IIS ExIS systems: connected with the VTrckS ExIS portal to submit vaccine orders, enroll provider master data, and submit wastage information. In return, VTrckS provided vaccine shipment data via downloads to jurisdictions’ IIS.
- Federal and commercial partners ExIS system called Vaccine Provider Ordering Portal (VPOP): VPOP was developed specifically for the COVID-19 vaccine program to support federal and commercial partners who lacked access to an electronic vaccine supply chain system capable of interfacing with VTrckS. As a web-based portal, VPOP allowed these partners to update delivery hours for receiving vaccine, order vaccines, and report inventory and wastage. It was also used by chain pharmacy and dialysis partners who had existing vaccine supply chain systems to ensure their standards aligned with VTrckS ExIS specifications since they had not exchanged vaccine data directly with CDC prior to the COVID-19 vaccination program.
- McKesson, CDC’s third-party vaccine distribution logistics provider: McKesson, which received and stored vaccine and ancillary supplies from manufacturers, received and filled provider orders based on data from VTrckS and shipped vaccine and ancillary kits. McKesson also communicated through VTrckS to transmit inventory information and shipment details to CDC.
- Federal data and reporting systems: The Immunization (IZ) Data Lake received data feeds from all CDC systems supporting the COVID-19 vaccine response, including VTrcks information on provider data, orders, and shipments.

The IZ Data Lake was developed for the COVID-19 vaccine program as a cloud-hosted data repository to receive, store, manage, and analyze deidentified COVID-19 vaccination data. CDC used the IZ Data Lake to store and process administration, enrollment coverage, logistics, vaccine product code, inventory, ordering, distribution, wastage, and provider data from CDC systems, jurisdictions, federal agencies, pharmacy partners, and dialysis partners. The IZ Data Lake also aggregated, analyzed, and provided data summaries and analytics to other reporting hubs, including for CDC COVID-19 Data Tracker and for Tiberius, a COVID-19 vaccine distribution planning, forecasting, tracking, modeling, and analysis application. [2]

Vaccine order requests from enrolled providers that were approved for shipment via VTrckS were then coordinated with manufacturers and distributors, and shipment information was communicated with the receiving vaccine providers. A schematic of vaccine distribution process is illustrated in Fig. 1.

## 4. Challenges For COVID-19 Vaccine Distribution and Tracking

Many challenges were unique to COVID-19 vaccine compared to the routine distribution of vaccines through the VFC Program and compared to the 2009 H1N1 pandemic response and other vaccine-related emergency responses. First, the large majority of COVID-19 vaccine doses administered in the U.S. were mRNA vaccines. These vaccines were the first mRNA vaccines ever utilized in the U.S., and one of the vaccines (Pfizer-BioNTech product) required ultracold storage, including during shipping. Because of the unique temperature requirements, the manufacturer developed special packing with dry ice and included electronic monitors so that each shipment could be electronically monitored en route. Dry ice supplies and an initial resupply of dry ice to maintain vaccine temperatures were made available to providers who did not have access to ultracold freezers and needed to utilize the specially designed packaging for temporary vaccine storage. Given the temperature requirements for shipping, special packing, and dry ice, VTrckS orders for Pfizer-BioNTech vaccine were sent to the manufacture who filled and shipped orders directly to providers, bypassing McKesson. (Fig. 1) Some of the resulting challenges associated with this arrangement included the need for providers and health departments to maintain awareness of different timelines for vaccine delivery, ordering cut off times, and cancellation procedures. CDC worked closely to align and coordinate the two types of distribution—central distribution and manufacturer distribution—and to proactively communicate with states and partners about the differences to facilitate their planning.

A further challenge for vaccines was the availability of multiple formulations that changed over time as did the mRNA vaccine storage and handling specifications. Over the course of the COVID-19 vaccine program, up to 8 different vaccine formulations were available at a time, with 5–10 doses per vial, and with different storage and handling requirements. Changes in doses per vial further complicated reporting of vaccine inventory and wasted doses. Moreover, time periods for storage of mRNA vaccines at 2–8 °C, a more typical storage temperature for routinely recommended vaccines, were incrementally extended over the course of the pandemic as data from manufactures and updated EUA approvals allowed. While a number of these changes represented maturation of the program, such as the addition of booster doses and pediatric formulations and the ability to store ultracold vaccine at refrigerated temperatures, the number and frequency of these changes added complexity for program participants.

As with the 2009 H1N1 pandemic, the COVID-19 vaccines were shipped concurrently with ancillary supply kits that included syringes, needles, and vaccine cards. Given worldwide demand for syringes and needles, and limited manufacturing capacity initially to expand production, obtaining appropriately sized needles and syringes to include in kits provided additional hurdles for U.S. Government logistics staff.

In addition to cold storage requirements, another challenge for providers was that the minimum order sizes for one of the two mRNA vaccines was initially very large (from 975 to 1170 doses) due in part to the special packaging and dry ice needed to ensure ultracold temperature requirements during shipping. In anticipation of larger-than-typical minimum order quantities, the CDC provider agreement allowed for redistribution of smaller quantities of vaccine. While redistribution helped to reduce wastage from expired vaccine, it created challenges with tracking of vaccination inventory and well as logistical challenges and resource requirements for state and local health departments and commercial partners.

Multi-dose vials were exclusively utilized by all manufacturers in order to improve vaccine manufacturing efficiency and increase access to vaccines as quickly as possible. However, particularly as vaccine demand waned, more vaccine wastage occurred. [26] (Fig. 3) Vaccine in multi-dose vials, once the vial is punctured, remain viable for a limited period of time. Thus, multi-dose vials contributed to wastage when insufficient numbers of people were available to utilize all doses in a vial while the vaccine remained viable. In addition, storage of mRNA vaccines at refrigerator temperatures also shortened shelf life, further contributing to vaccine wastage.

Other challenges to successful distribution of vaccines included typical weather-related incidents impacting cold chain deliveries, including hurricanes and blizzards. Staff turnover and significant variability in vaccine demand over time also created challenges assuring appropriate management of the many parts of the COVID-19 vaccination distribution system.

## 5. Provider recruitment and training

Because COVID-19 vaccines were first approved for persons 16 years and older or 18 years and older, many VFC providers did not initially participate in the program. In addition, providers may have declined participation because they were unable or unwilling to comply with the greater reporting requirements for COVID-19 vaccine providers.

Recruitment of providers and onboarding them into state IIS systems were highly time intensive tasks for state and local health departments and required ongoing investments to support providers with questions and challenges related to required reporting. In addition, given the newness of working with a vaccine requiring ultracold storage and changing storage and handling requirements for COVID-19 vaccines over time, additional investments were needed to provide frequent updated communications and ongoing training opportunities.

## 6. Constantly Changing Landscape

**Fig. 2.**
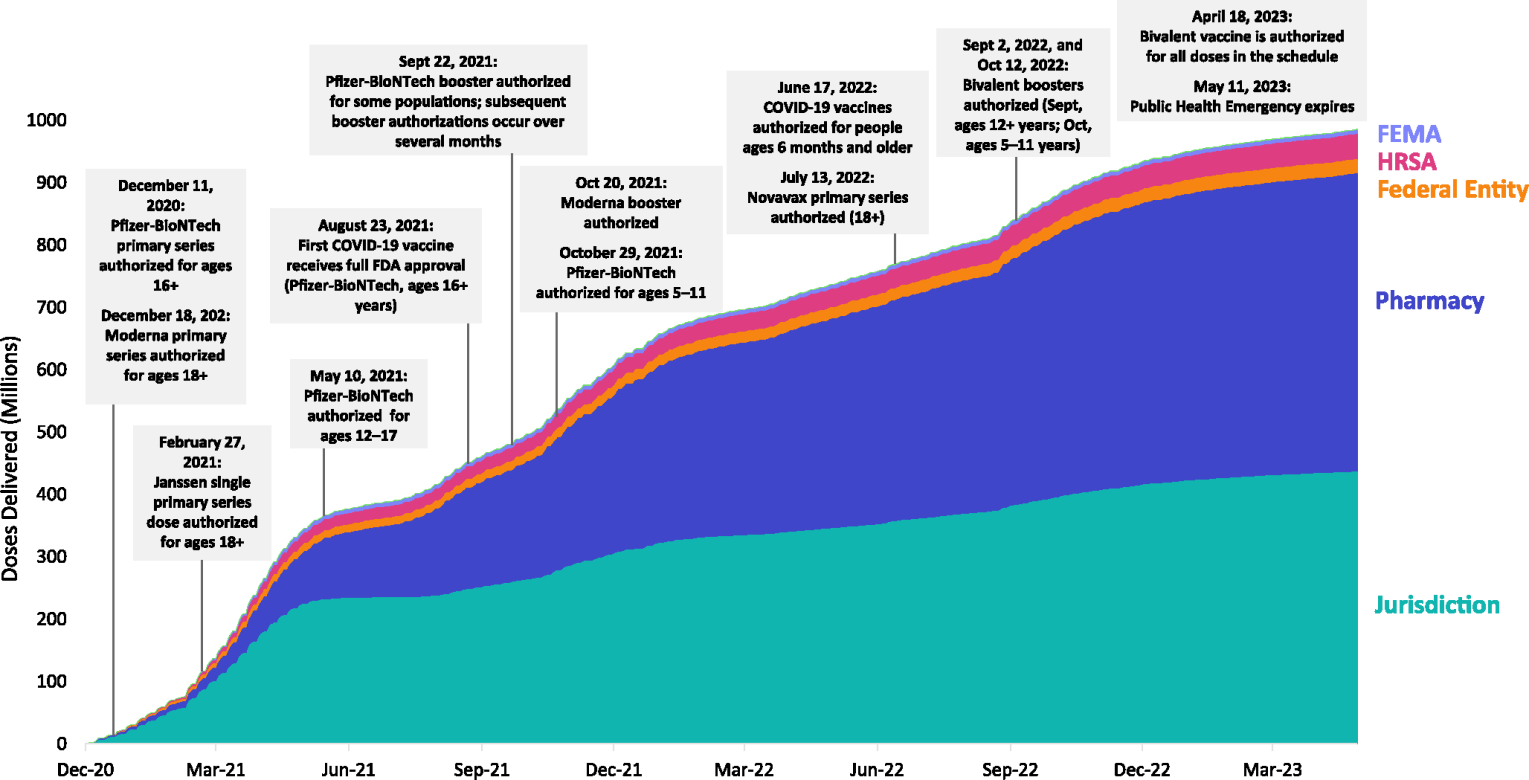
COVID-19 vaccine program milestones and COVID-19 vaccine doses distributed between December 1, 2020 and May 11, 2023. Footnote: As of May 11, 2023, the total number of doses distributed to each of the entities were 615,810 to dialysis partners (data not shown), 6,232,490 to Federal Emergency Management Agency (FEMA) partners, 40,047,825 to Health Resources and Services Administration (HRSA) partners, 23,100,795 to other federal entities, 478,221,785 to pharmacies, and 437,580,445 jurisdiction-enrolled providers.

The distribution system, public health, and providers were challenged with frequent updates in vaccine EUA changes, changes in vaccine storage and handling, updated clinical guidance, and multiple incremental steps of expanding vaccine use for children. Although the changes made in mRNA vaccines by manufacturers over time led to multiple updates, the changes also represented important improvements in providers’ ability to store and administer COVID-19 vaccines. In addition, safety concerns regarding Johnson and Johnson’s Janssen COVID-19 vaccine led to a relatively abrupt decline in its use. [27] Delays in the availability of the Novavax vaccine in the vaccine program relative to the mRNA and Janssen vaccines further complicated the vaccine supply landscape. Timing of changes in recommendations and other events relative to vaccine distribution are illustrated in Fig. 2.

Multiple ongoing outreach efforts to state and local health departments, vaccine providers, and other vaccine partners were conducted to give regular updates regarding changes, offer technical support, and offer opportunities to ask questions. The CDC COVID-19 vaccine distribution team hosted on-line office hours up to 3 times per week early in the vaccination program and met individually with states as needed; gave updates for providers and vaccine partners via webinars; developed and distributed a weekly newsletter that included changes in formulations, ordering processes and procedures, and provided updates on vaccine supply timeliness and delivery delays for holidays and weather-related concerns. Implementation of these communications strategies were paramount to keeping providers and partners and other collaborators up to date on the many changes that impacted their access to COVID-19 vaccines and aided their planning for vaccine administration at the local level.

## 7. Overall Vaccine Distribution Milestones During the first year of the U.S. COVID-19 vaccine program (December 2020 through December 2021)

The most intensive vaccine distribution efforts occurred during year one of COVID-19 vaccine availability when vaccine demand was at its highest. [27–28]

- 723,566,095 vaccine doses were shipped
- 97,000 providers were directly shipped vaccine
- 1,312,967 vaccine shipments were made
- 391,567,275 doses shipped with dry ice orders
- 664,422,865 doses shipped with ancillary kits
- 521,797,692 doses were administered
- 244,104,717 people had at least one dose, and
- 210,360,591 people had completed a vaccine primary series, including 89 % of all persons 65 years of age and older, the group at highest risk of severe disease and death.

**Fig. 3.**
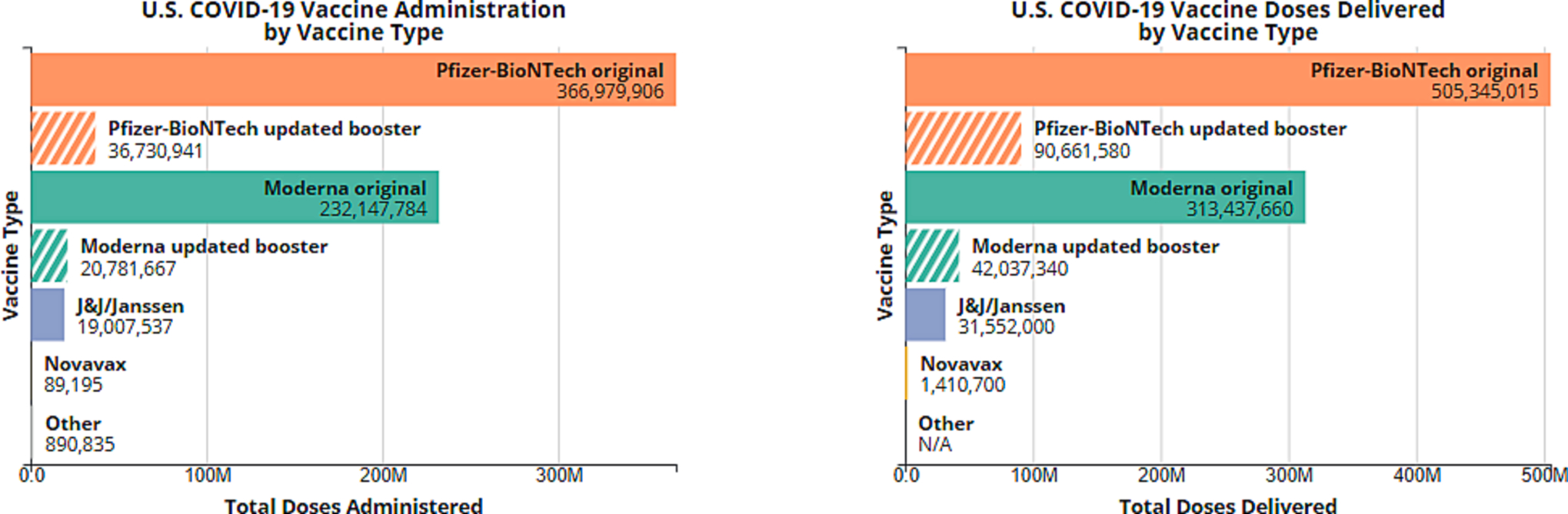
U.S. COVID-19 vaccine doses administered and delivered by vaccine manufacturer and vaccine type (monovalent original formulation and bivalent updated formulation) between December 2020 and May 11, 2023. Footnote: “Other” includes vaccines administered outside of the United States that were not vaccine brands that were included in the U.S. purchase of vaccines. All COVID-19 vaccines available for administration in the U.S. were federally purchased and could not be privately purchased during the health emergency. For the data timeframe “Updated booster” refers only to the bivalent COVID-19 vaccine.

By the end of the COVID-19 pandemic response emergency declaration on May 11, 2023, 984,444,295 total doses had been distributed, 676,728,782 doses had been administered, and 81.4 % of the U.S. population had received one or more doses of a COVID-19 vaccine. These totals include 139,918,910 doses of updated bivalent COVID-19 mRNA vaccine distributed, and 17 % of the U.S. population that had received at least one updated bivalent COVID-19 vaccine dose. [28] (Fig. 3)

## 8. Conclusions And Lessons Learned

COVID-19 vaccine distribution presented many new challenges compared to prior large-scale vaccination efforts. These challenges, however, were met, in part, due to lessons learned from prior experiences and planning; leveraging existing infrastructure in place for VFC; utilizing existing standards and processes; standing up new systems where needed; and, dedicated collaboration, partnership, and problem solving with state and local health departments, distributors, manufactures, multiple federal agencies, and vaccine providers. A key to successful collaborations across partners in the delivery of vaccines to end users was communication frequently and in multiple formats (e.g., newsletters, office hours, etc.), as noted above, to provide opportunities for discussion, clarifications, and reminders about storage and handling, shipping, inventory management, and other issues. Enhancing IT infrastructure to improve information exchanges regarding vaccine distribution, use, supply and wastage information led to substantially greater visibility of vaccine supply and administration data and information for data-driven decision making. Enhancements made to existing infrastructure utilized for VFC, IIS, and VTrckS; creating VPOP for federal and commercial partners; and making enhancements to Vaccine Finder (aka Vaccines.gov) not only were essential to successful distribution of almost one billion COVID-19 vaccine doses, but also have improved readiness for future vaccine-related emergency responses. The COVID-19 vaccine program also highlighted the importance of continuously updating systems and updating routine vaccine providers’ and partners’ connections to these systems in order to reduce the burden of connecting providers to systems during vaccine-related emergencies. Continued improvements in the utilization IIS for routine immunizations among those providing vaccines for adults and children will also improve readiness for future outbreaks and pandemics. Limited surge capacity for manufacturing of needles and syringes also reinforced the need to consider these supply issues early as part of emergency vaccine development and distribution planning.

Despite multiple challenges, lifesaving COVID-19 vaccines were made widely available, minimizing vaccine access challenges for people of all ages in communities across the United States.

## Data Availability

No data was used for the research described in the article.
